# Supplementary material for: The Ral small GTPase is an essential regulator of Exocyst complex function in secretion
Source: bioRxiv. 2025 Aug 31:2025.08.28.672893. Preprint. [Version 1] doi: 10.1101/2025.08.28.672893 (PMC12407918; doi:10.1101/2025.08.28.672893)
Supplement: Supplement 1 [file NIHPP2025.08.28.672893v1-supplement-1.pdf]

Wu Figure S1

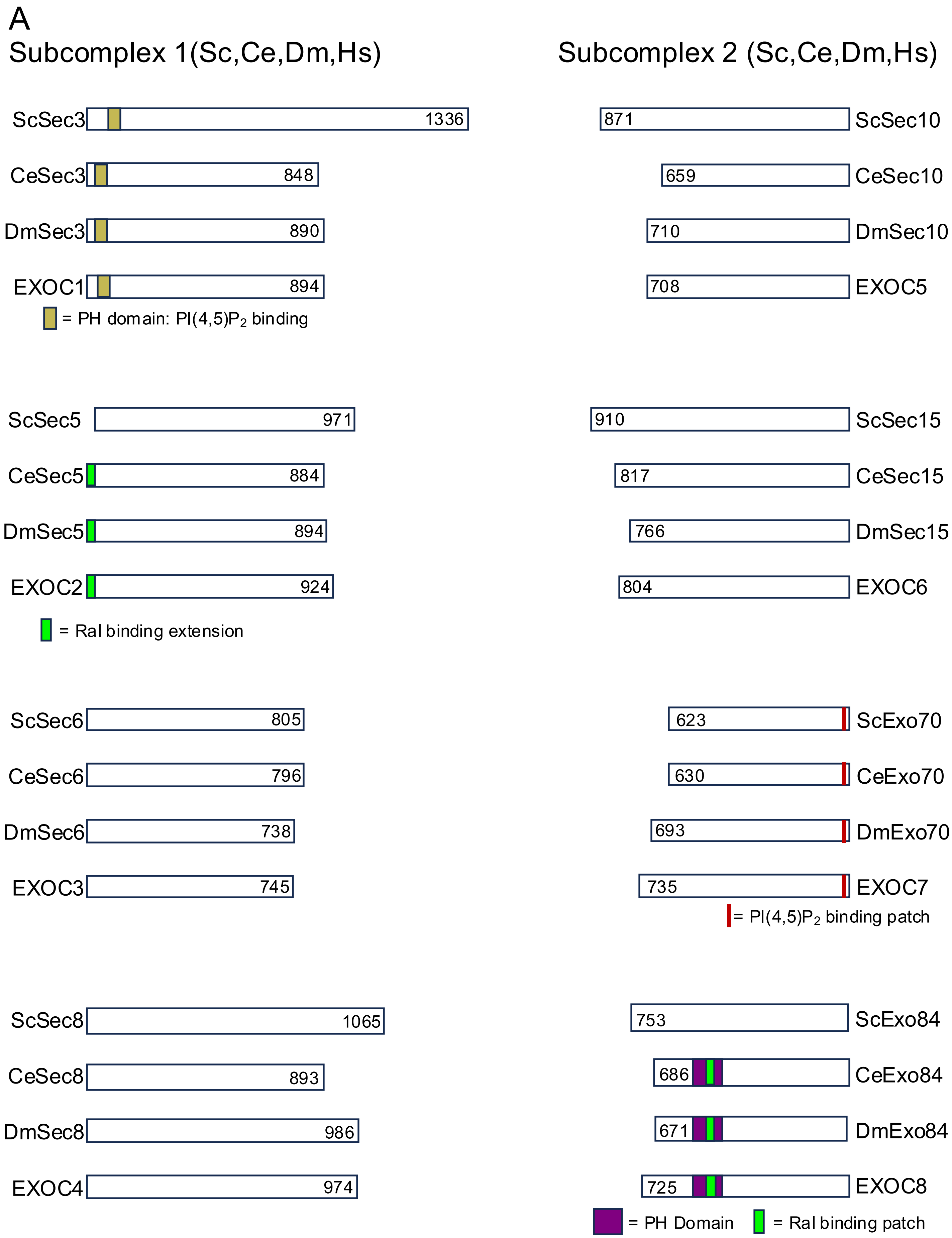
A
Subcomplex 1(Sc,Ce,Dm,Hs)
Subcomplex 2 (Sc,Ce,Dm,Hs)
ScSec3
1336
CeSec3
848
DmSec3
890
EXOC1
894
= PH domain: PI(4,5)P2 binding
871
ScSec10
659
CeSec10
710
DmSec10
708
EXOC5
ScSec5
971
CeSec5
884
DmSec5
894
EXOC2
924
= Ral binding extension
910
ScSec15
817
CeSec15
766
DmSec15
804
EXOC6
ScSec6
805
CeSec6
796
DmSec6
738
EXOC3
745
623
ScExo70
630
CeExo70
693
DmExo70
735
EXOC7
= PI(4,5)P2 binding patch
ScSec8
1065
CeSec8
893
DmSec8
986
EXOC4
974
753
ScExo84
686
CeExo84
671
DmExo84
725
EXOC8
= PH Domain
= Ral binding patch

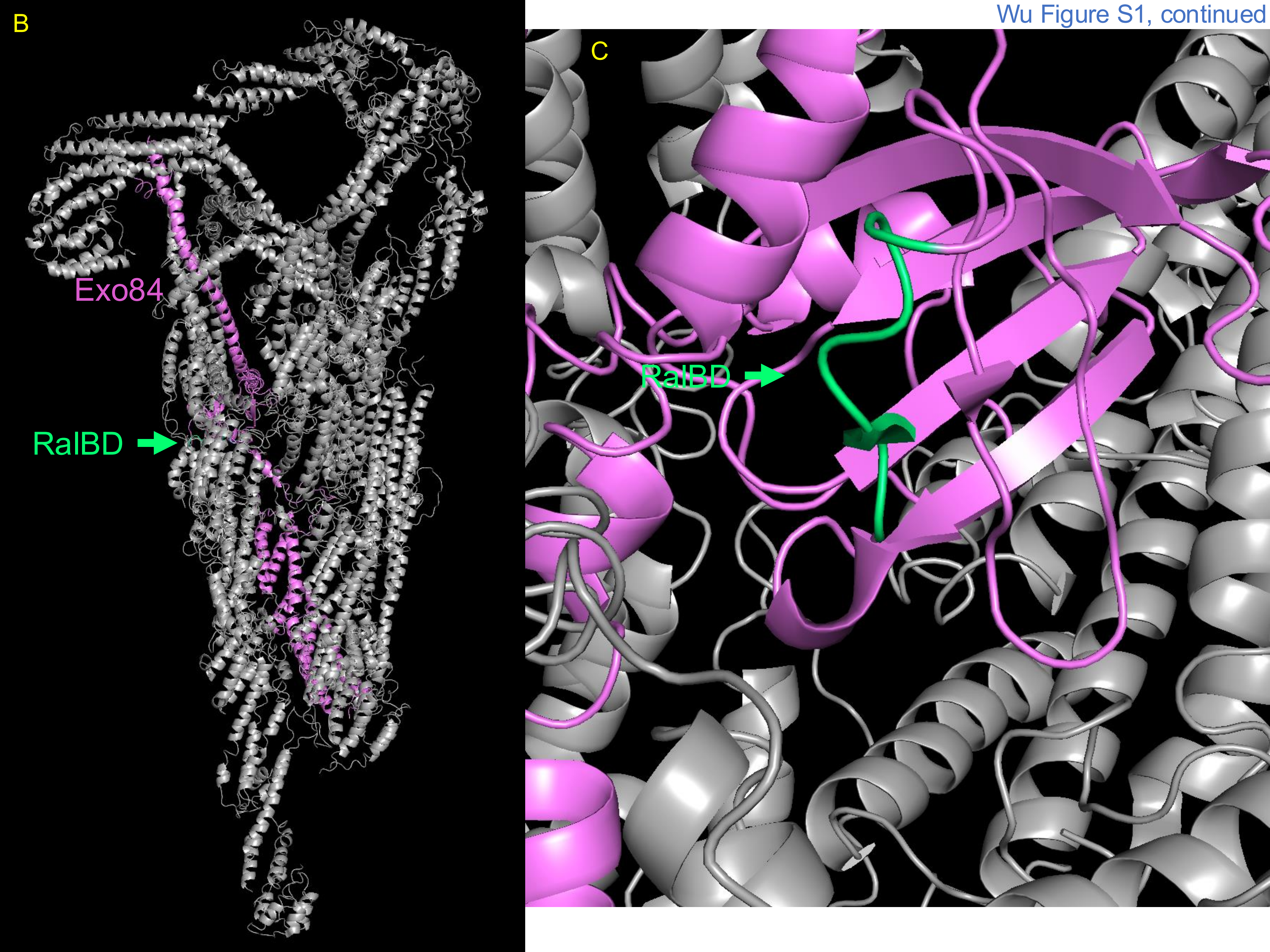
B
Exo84
RalBD
C
RalBD

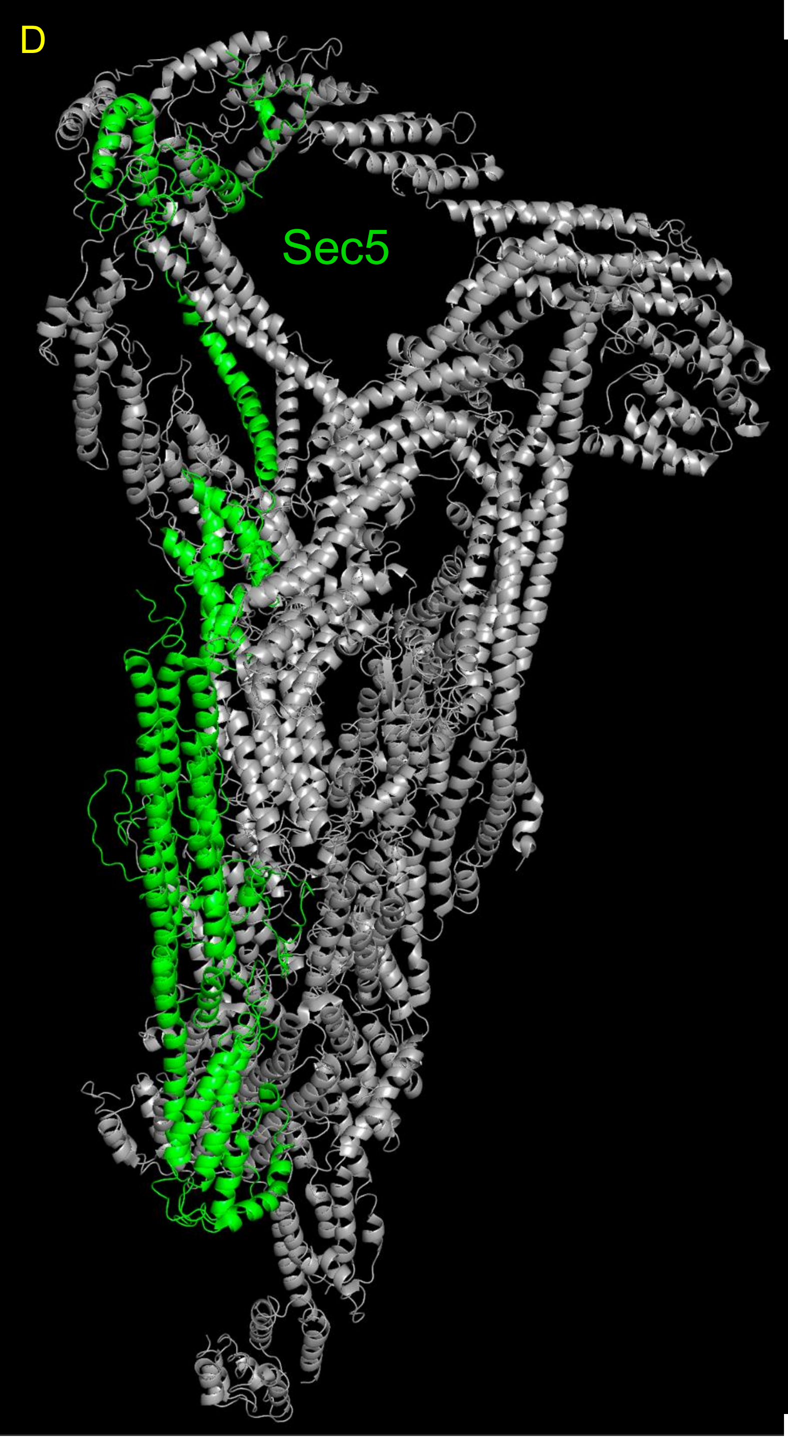
D
Sec5

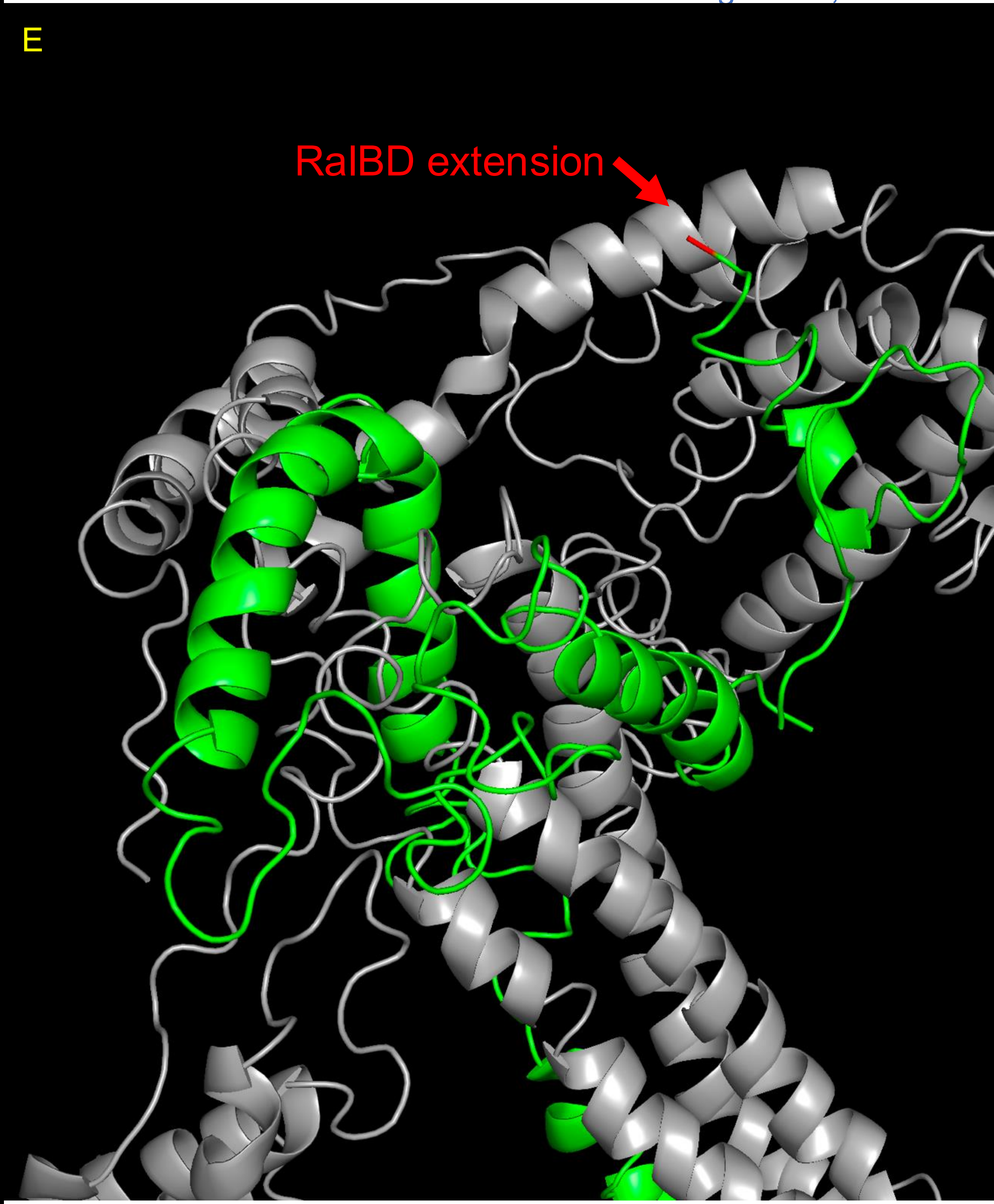
E
RalBD extension

Wu Figure S1, continued

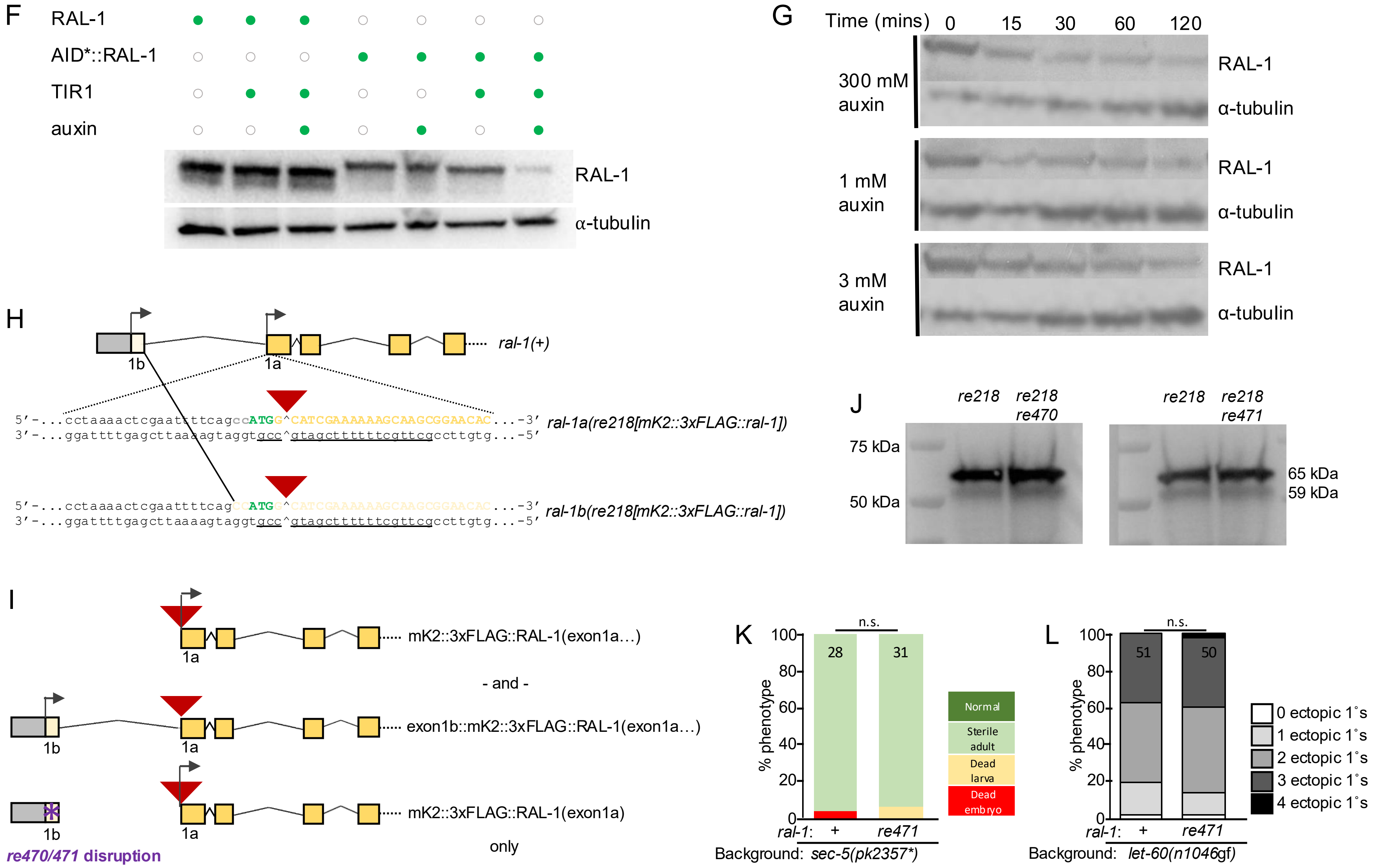

**Figure S1: Ral, the Exocyst complex, and conserved sequence domains and motifs in the eight Exocyst subunits from four species. (A)** *S. cerevisiae* (Sc), *C. elegans* (Ce), *D. melanogaster* (Dm), and *H. sapiens* (Hs), divided into Subcomplex 1 and Subcomplex 2. The Exocyst is a CATCHR (complexes associated with tethering containing helical rods) family of multisubunit tethering complexes (MTCs) used in vesicular trafficking and fusion. Exocyst subunits do not contain catalytic domains. Rather, all of them are composed primarily of alpha helices with limited regions of binding interfaces. Many of these alpha helices are organized into helical bundles. Each subunit in turn is assembled into pairs with its partner (Sec3-Sec5, Sec6-Sec8, Sec10-Sec15, Exo70-Exo84), the partners are assembled into Subcomplexes 1 and 2 (Sec3/5/6/8 and Sec10/15/Exo70/84, respectively), and the two subcomplexes assembled into the heterooctameric mature complex (Mei *et al.* 2018). Consequently, the helical bundles in part govern complex assembly and are thus distinctive for each subunit: the are distinctive and the sequence and structural levels and can be identified among orthologs of a subunit across evolution. For example, the Pfam Sec3 domain is poorly conserved at the level of primary sequence but is readily identified through sequence analysis programs like SMART, used here. The eight Pfam domains of helical clusters, one for each subunit, are not shown in this figure, but are shown in Appendix 1 for this study. This Appendix provides all the sequence files and alignments where domains of each protein are annotated and alignments of each subunit family are collated. Outside of helical bundles, the conventional domains of the Exocyst are shown in this schematic: an unconventional PH domain in Sec3 validated in yeast as binding the phospholipid PI(4,5)P, and an unconventional PH domain in Exo84 that no longer binds lipids (and is absent in yeast). Studies of Ral have implicated two binding sites, in Exo84 and Sec5.These were originally identified via yeast two hybrid and validated with fragment binding *in vitro* and in cultured cels. The fragments of mammalian subunits have been crystalized with human RALA (Fukai 2003; Jin 2005). The Sec5 fragment is an N-terminal extension present in metazoans but not in yeast. The Exo84 fragment includes a non-functional mid-protein PH domain, potentially evolved to support Ral binding. The corresponding putative Ral-binding sequence in Exo84 is not conserved in yeast. Alignments of both are shown in **Fig. S8C**.

Subcomplex 1 subunits:
Sec3: Uniprot #s: P33332, Q20678, Q9VVG4, Q9NV70
Sec5: Uniprot #s: P89102, Q22706, Q9VQQ9, Q96KP1
Sec6: Uniprot #s: P32844, Q19262 , Q9V8K2, O60645.
Sec8: Uniprot #s: P32855, Q9XWS2, Q9VNH6, Q96A65.

Subcomplex 2 subunits:
Sec10: Uniprot #s: Q06245, Q18406, Q9XTM1, O00471
Sec15: Uniprot #s: P22224, Q18286, Q9VDE6, Q8TAG9
Exo70: Uniprot #s: P19658, P91149, Q9VSJ8, Q9UPT5
Exo84: Uniprot #s: P38261, Q95Q35, Q9VBI4, Q8IYI6

Pfam domains for each Exocyst subunit: detectable by sequence-based algorithms, structural conservation, shared across orthologous proteins, thought to be a core complex module

**Figure S1, continued. (B)** A model of yeast Exocyst complex structure derived from Cryo-EM (Mei *et al.* 2018). Structure was analyzed using PyMOL 3.1.6.1. Exo84 is colored pink. The Pfam Exo84 domain, organized into helical bundles, is evident at the bottom of the model (see Appendix for annotated individual sequences for all four species for Exo84, at the C-term, bottom here, but also for other subunits. **(C)** A zoom to see the loop corresponding to the Ral-binding domain (RalBD) of Exo84, highlighted and indicated by an arrow in blue-green. This loop is present in yeast Exo84, of which this is a model, but the sequence is only that of the RalBD in metazoans (Appendix; **Fig. S1A Fig. S8C**). **(D)** The same model based on CryoEM model of the Exocyst, rotated 180˚ on its long axis, with Sec5 colored lime green. **(E)** A zoom to see the N-terminus of Sec5 at the top of the model, with the N-terminal methionine colored red for orientation. Metazoans encode an N-terminal extension at this point, not found in yeast, that is thought to contain another RalBD (Appendix; **Fig. S1A Fig. S8C**). **(F)** Immunoblot validation of chemical genetic depletion of AID*::mKate2::3xFLAG::RAL-1: animals of genotype *ral-1(re218[mKate2::3xFlag::ral-1])* or *ral-1(re218re319[AID*::mKate2::3xFlag::ral-1])* singly or in combination with *ieSi57[eft-3p>TIR1::mRuby]*, with or without addition of auxin (IAA = indole acetic acid) and immunoblotted with α-FLAG antibody. The upward band shift between the first three and the latter three lanes is due to the addition of AID*. **(G)** Immunoblot detection of time course of auxin addition and different concentrations indicates that depletion of RAL-1 is incomplete regardless of dose and time. This may be due to inaccessibility of the AID* tag to TIR1-auxin when bound in complexes, specifically the Exocyst, or perhaps poor accessibility of the AID* target in the context of tagged RAL-1. **(H)** Note the two RAL-1 bands variably visible in most exposures. In our previous study (Shin *et al.*, 2018, Fig. S6), we hypothesized that the upper band is the predicted 61 kDa RAL-1B isoform with a non-canonical-length N-terminal extension unique to Nematoda, while the lower band is the 56 kDa RAL-1A isoform with a canonical-length N-terminal extension present in most metazoans. The alternative splicing of the upstream exon 1b and canonical exon 1a are shown: both are predicted to splice into the RAL-1 tags: AID*::mK2::3xFLAG or mK2::3xFLAG tag and thus be detectable by western blot. **(I)** We selectively disrupted exon 1b with CRISPR-based STOP-IN (Wang, 2018), generating alleles *re218re470* and *re218re471*. We evaluated the function of deficient exon 1b animals in the two known activities of *ral-1*: exocyst and signaling. **(J)** Neither *re470* nor *re471* altered expression of tagged endogenous RAL-1 as detected by α-FLAG immunoblotting. Since by RNAseq RAL-1A is predicted to be much more abundant than RAL-1B (see Results), RAL-1A may mask RAL-1B protein as well as the consequences of disrupting exon 1b. Bands sizes remain mysterious. The 65 kDa band corresponds to unmodified RAL-1B or perhaps post-translationally modified RAL-1A. The much less abundant 59 kDa band corresponds to the expected size of unmodified RAL-1A. **(K)** *re471* did not alter Exocyst function in animals deficient for M+Z- *sec-5 (*this study) or **(L)** induction of 2˚ VPC fates as assayed by antagonism of *let-60(n1406*gf*)* induction of ectopic 1˚ VPCs (our other studies). We conclude that, in addition to be an apparently nematode-specific alternative exon of *ral-1*, exon 1b is not required for established functions of *ral-1* in Exocyst or signal transduction.

Wu Figure S2

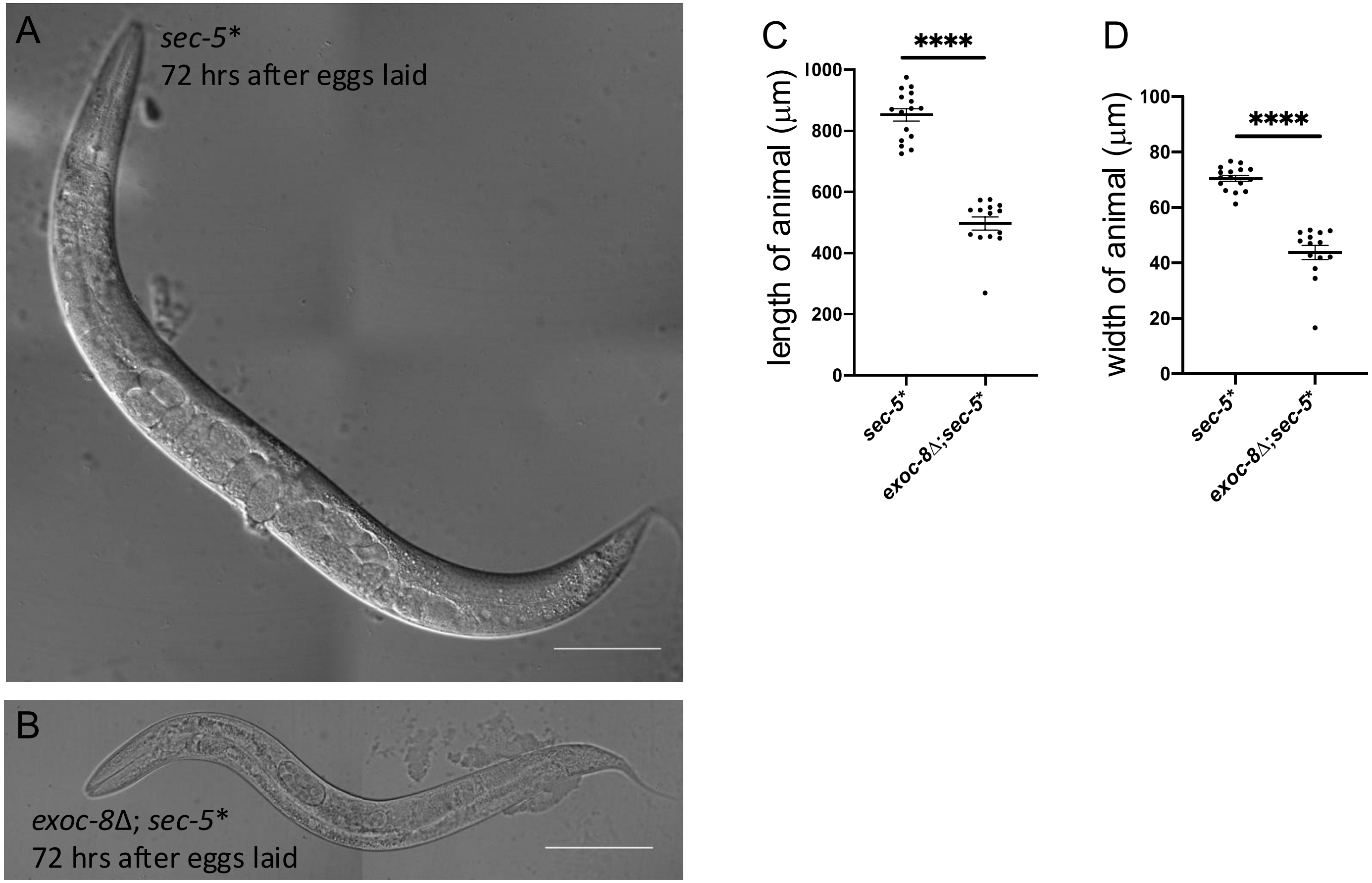
A
sec-5*
72 hrs after eggs laid
B
exoc-8Δ; sec-5*
72 hrs after eggs laid
C
length of animal (μm)
1000
800
600
400
200
0
****
sec-5*
exoc-8Δ;sec-5*
D
width of animal (μm)
100
80
60
40
20
0
****
sec-5*
exoc-8Δ;sec-5*

Wu Figure S2

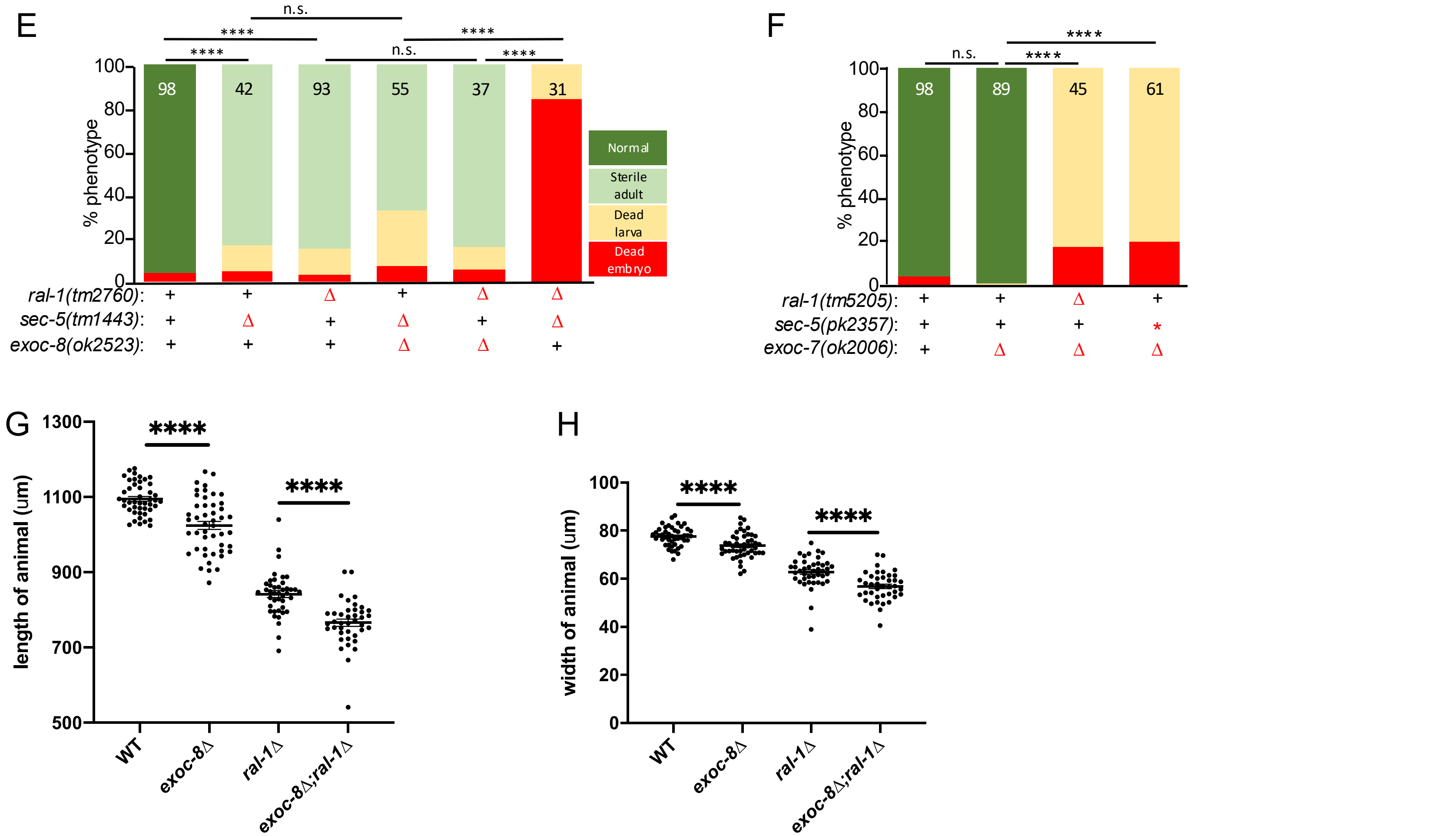


**Figure S2. Combining reduction-of-function mutations among *ral-1* and Exocyst components aggravates growth/developmental effects. A-D)** *exoc-8(ok2523)*; *sec-5*(*pk2357**) double mutants from **Fig. 1C** did not die at earlier larval stages but were substantially decreased in length **(C)** and width (**D**), which could be superficially mistaken for earlier lethality. **(A,B)** Representative DIC photomicrographs of synchronized *sec-5*(*pk2357**) **(A)** and *exoc-8*(*ok2523*); *sec-5*(*pk2357*) **(B)** animals. **(E,F)** Genetic interactions among second alleles of *ral-1*, *sec-5* and non-essential subunit-encoding *exoc-7* in support of **Fig. 1C**. Color-coded genotype key is right of the two graphs. **(E)** Genetic interactions with second alleles *ral-1(tm2706)* (Zand *et al*., 2011) and *sec-5(tm1443)* with the *exoc-8(ok2523)* allele used in **Fig. 1C**. **(F)** *sec-5* and *ral-1* alleles used in **Fig. 1C** but in combination with deleted *exoc-7*, which encodes the Exo70 non-essential subunit reported to dimerize with Exo84 and assemble with Subcomplex II (Mei 2018; LePore 2018). **(G,H)** *exoc-8(ok2523)* and *ral-1*(*tm5205)* single and double mutants from **Fig. 1C** did not die at earlier larval stages but manifested substantially decreased length (**G**) and width (**H**), which could be superficially mistaken for earlier lethality. Genotype key is below each graph. ****<0.0001, n.s. = not significant (Fisher's exact test). Scale bar = 100 μm

# Wu Figure S3

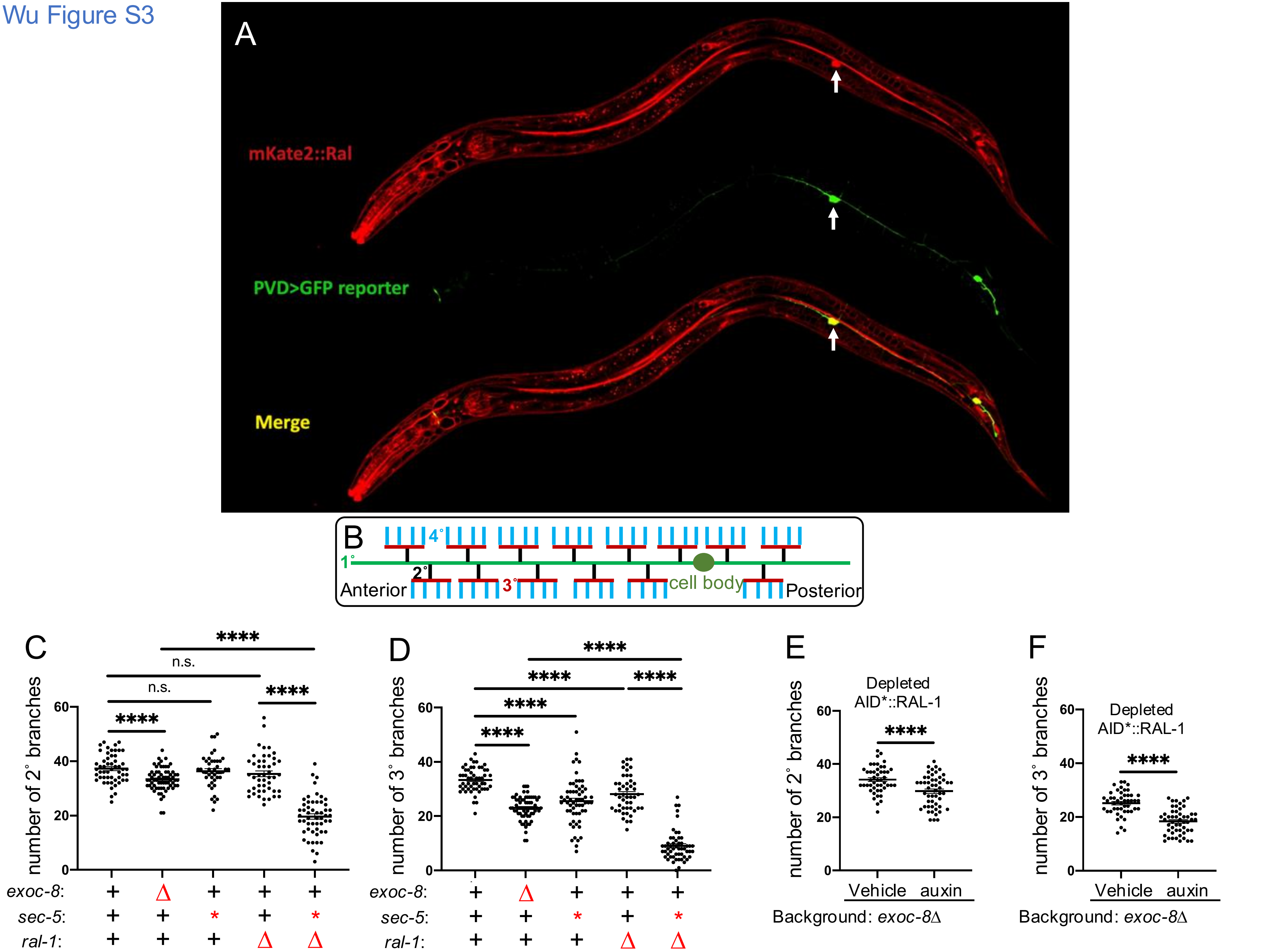
A
mKate2::Ral
PVD>GFP reporter
Merge
B
4°
1°
2°
3°
Anterior
cell body
Posterior
C
D
E
F
****
n.s.
number of 2° branches
number of 3° branches
exoc-8:
sec-5:
ral-1:
Depleted AID*::RAL-1
Vehicle
auxin
Background: exoc-8Δ

**Figure S3. RAL-1 is required for full exocyst-dependent PVD dendritic arborization. (A)** Maximum intensity projection of z-series confocal photomicrographs of endogenous mKate2::3xFLAG::RAL-1 protein colocalized with GFP-labelled (*wdIs52[ser-2(prom3)p>gfp]*)) cell body of the PVD neuron (arrows); red = RAL-1, green = PVD label, yellow = merge. **(B)** A schematic of the 1˚ (green), 2˚ (black), 3˚ (red) and 4˚ (blue) branches of the PVD neurons. Anterior is left for both animals. **(C,D)** The *exoc-8(ok2523)*; *ral-1(tm5205)* double mutants exhibit more severe defects in 2˚ **(C)** and 3˚ **(D)** dendritic branching than each single mutant. **(E,F)** Animals of genotype *ieSi57[eft-3p>TIR1::mRuby]*; *ral-1(re218re319[AID*::mKate2::3xFLAG::ral-1])* display auxin-dependent enhancement of the 2˚ and 3˚ dendritic branching defect conferred by *exoc-8*(*ok2523*). ****<0.0001, n.s. = not significant (*t*-test).

Wu Figure S4

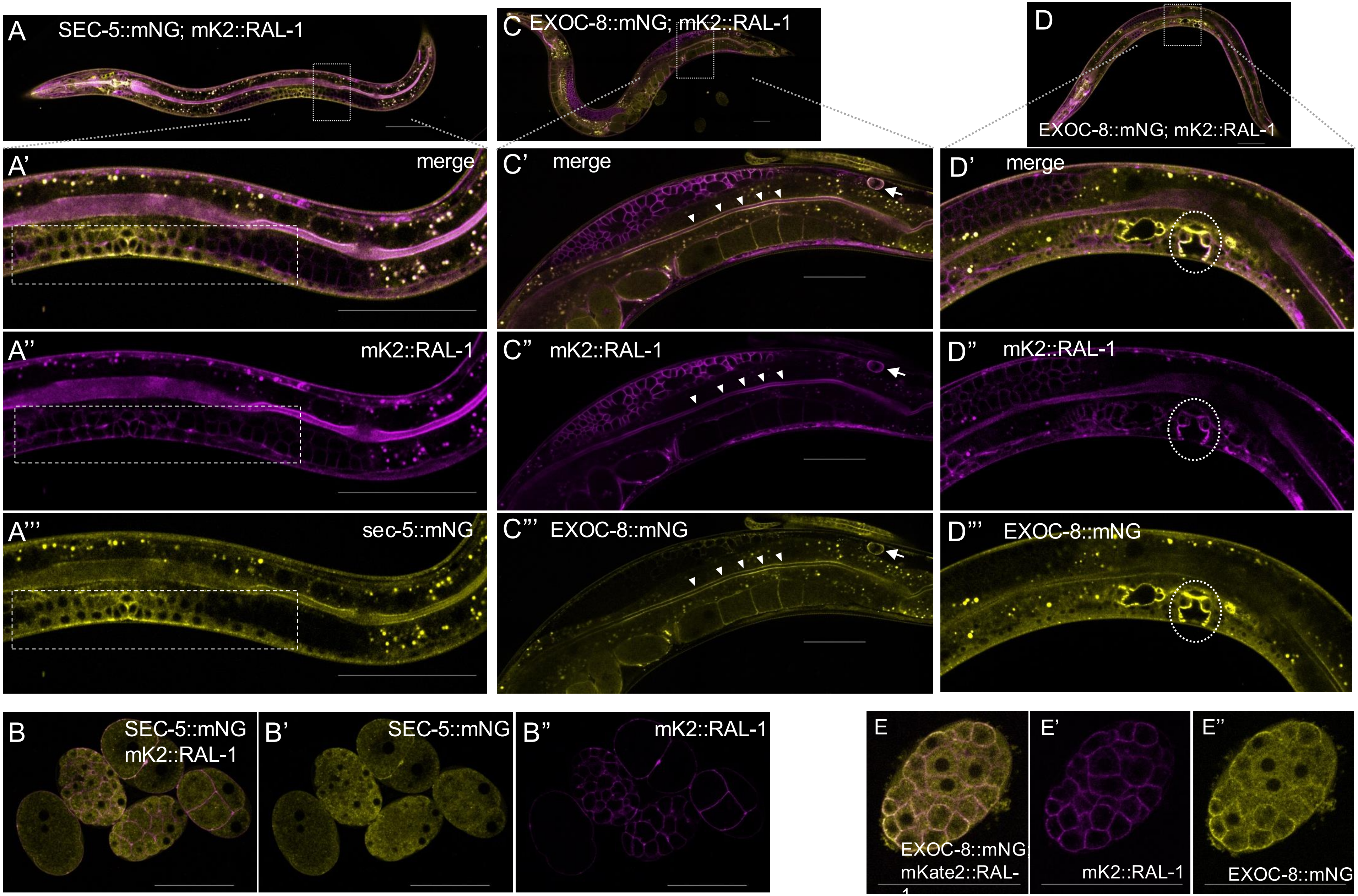
A
SEC-5::mNG; mK2::RAL-1
C
EXOC-8::mNG; mK2::RAL-1
D
EXOC-8::mNG; mK2::RAL-1
A'
merge
C'
merge
D'
merge
A''
mK2::RAL-1
C''
mK2::RAL-1
D''
mK2::RAL-1
A'''
sec-5::mNG
C'''
EXOC-8::mNG
D'''
EXOC-8::mNG
B
SEC-5::mNG
mK2::RAL-1
B'
SEC-5::mNG
B''
mK2::RAL-1
E
EXOC-8::mNG;
mKate2::RAL-1
E'
mK2::RAL-1
E''
EXOC-8::mNG

**Figure S4:** Whole-animal **(A)** and zoomed spinning disk confocal photomicrographs of merged **(A')**, magenta **(A'')** and yellow **(A''')** spinning disk confocal photomicrographs of mKate2::RAL-1 with SEC-5::mNG colocalized on the plasma membrane of VPC and vulval precursor cells (VPCs; dotted line rectangle) of a late L3 animal. **B-B'')** The same for a group of embryos. **C-C''')** Whole animal **(C)** and zoomed images of of merged **(C')** magenta **(C'')** and yellow **(C''')** spinning disk confocal photomicrographs mKate2::RAL-1 with EXOC-8::mNG colocalized on the plasma membranes of a coelomocyte (arrow) and apical brush border of intestinal cells (solid triangles) of an adult animal, **D-D''')** on the apical membranes of invagination 22 VPC lineage progeny (dotted circle), and **E-E''')** on the plasma membranes of an embryo. Scale bars = 50 µm.

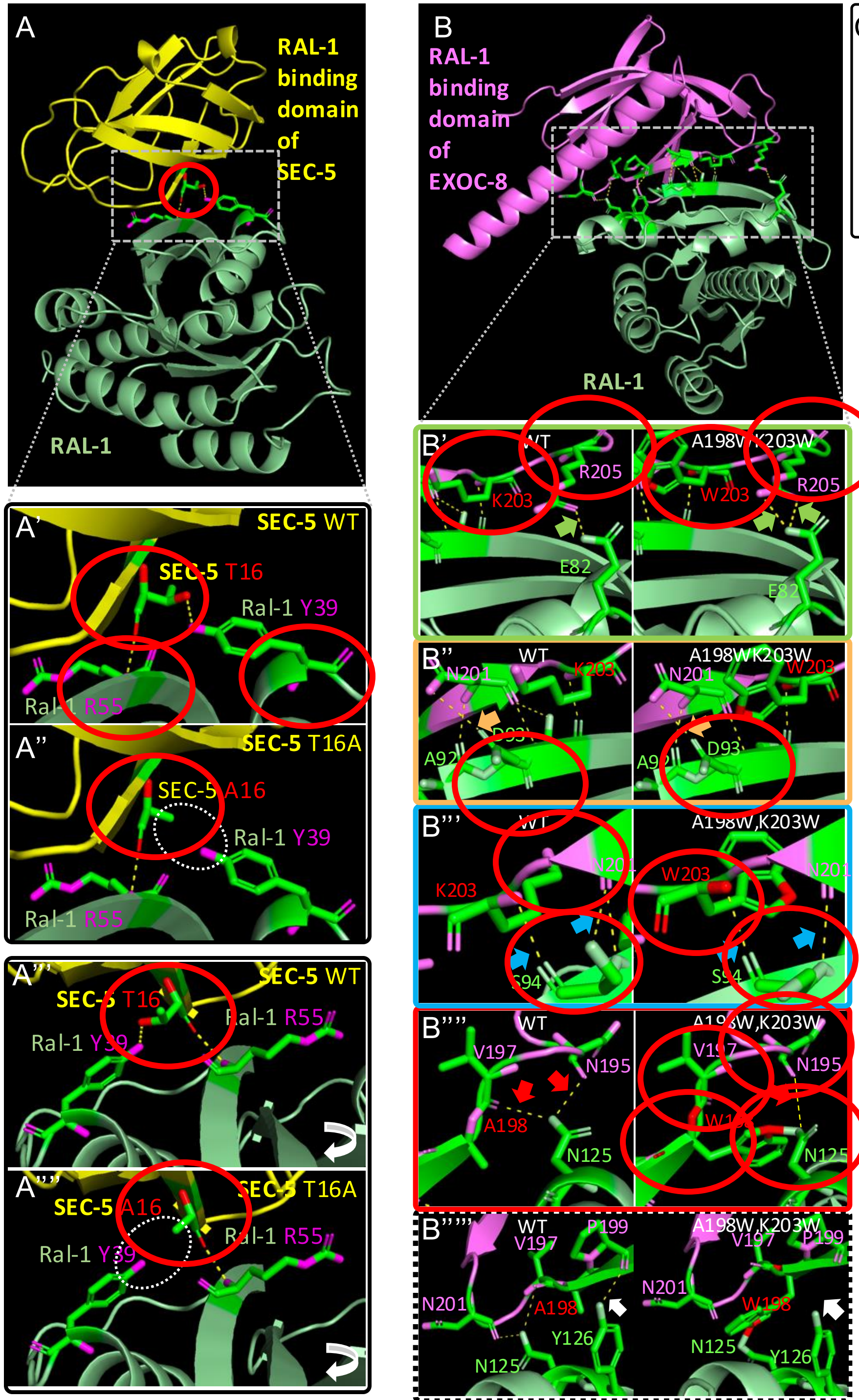


C
```
Hs SEC5     1 -----MSRSRQPPLVTGISPNEGIPWTKVTIRGENLGTGPTDLIGLTICG 45
Dm SEC5     1 --------MAPQPVVTGLSPKEGPPGTRVIIRGEFLGTRVQDLIGLKICG 42
Ce SEC-5    1 MEENAQARERLPPTVTGLSPTEGVPGTQITIRGENLGNDQSDVIMLFICG 50
                             *  ***:**.**:*  *::  ****  **.    *:*  *  ***

Rn EXO84   216 YRYNALYPLDRLAVVNVKDNPP----MKDMFKLLMFPESRIFQAENAKIK 261
Dm EXO84   178 LDFLTEYDPKKIAVINIKDLDG----VKNAINIITPDGSKIYQSITAAGK 223
Ce EXOC-8  186 YVMESTLSLNSVAPVNVKDRESGAANAGKVLKLLIFPESRCYLCESARIR 235
                    :      ..:*  :*:**          .  ::::      *:  :  .  .*   :
```

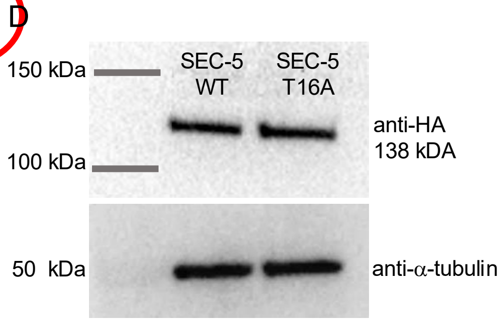


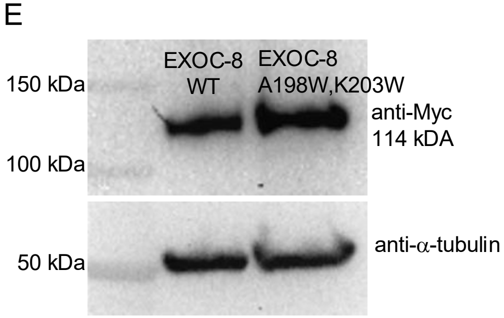


F

| RAL-1 residue | EXOC-8 WT residue | Distance (Å) | EXOC-8 A198W,K203W residue | Distance (Å) |
|---|---|---|---|---|
| GLU 82 O | ARG 205 N | 2.5 | ARG 205 N | ↑2.77 |
| GLU 82 O | ARG 205 N | / | ARG 205 N | 2.89 |
| ASP 93 O | ASN 201 N | 3.35 | ASN 201 N | ↓3.15 |
| SER 94 O | LYS 203 N | 2.74 | TRP 203 N | ↑2.82 |
| SER 94 O | ASN 201 O | 3.54 | ASN 201 O | ↓2.69 |
| SER 94 N | ASN 201 O | 2.77 | ASN 201 O | / |
| ASN 125 N | VAL 197 O | 3.24 | VAL 197 O | / |
| ASN 125 N | ASN 195 O | 2.89 | ASN 195 O | ↑3.29 |
| TYR 126 O | PRO 199 O | 3.07 | PRO 199 O | / |

Wu Figure S5

**Figure S9: Structural models of Ral-effector interface mutations of SEC-5 and EXOC-8. A)** Alphafold2 predicted model of SEC-5 interaction with RAL-1. With zoom-in photos **(A')** and rotated angle **(A'')** of WT SEC-5 (upper photos **A'** and **A''**) and T16A mutant SEC-5 (lower photos of **A'** and **A''**) and RAL-1 interaction domains. **B,F)** alphafold2 predicted model of EXOC-8 interaction with RAL-1. Zoom in photos are WT (**B'-B""'** left photos) vs. A198W,K203W mutant EXOC-8. Color arrows pointing at changed polar contact distance, matches Table in **F**. **C)** Alignment of SEC-5 T16 flanking sequences (upper) and EXOC-8 A198, K203 flanking sequences of *C. elegans* (CE), homo sapiens (Hs) and Drosophila melanogaster **(D)**. **D,E)** T16A mutation of SEC-5 **(D)** and A198WK203W of EXOC-8 **(E)** did not destabilized protein showed by western blot.
